# BiVO_4_–Deposited MIL–101–NH_2_ for Efficient Photocatalytic Elimination of Cr(VI)

**DOI:** 10.3390/molecules28031218

**Published:** 2023-01-26

**Authors:** Huiwen Sun, Qihang Dai, Ju Liu, Tiantian Zhou, Muhua Chen, Zhengchun Cai, Xinbao Zhu, Bo Fu

**Affiliations:** Jiangsu Co-Innovation Center of Efficient Processing and Utilization of Forest Resources, Jiangsu Provincial Key Lab for the Chemistry and Utilization of Agro-Forest Biomass, College of Chemical Engineering, Nanjing Forestry University, Nanjing 210037, China

**Keywords:** photocatalyst, hexavalent chromium, Z–scheme heterojunction, charge separation efficiency

## Abstract

In this study, a flower–like BiVO_4_/MIL–101–NH_2_ composite is synthesized by a facile and surfactant–free process. The –COO^−^–Bi^3+^ ionic bond construction was conductive to enhance the interface affinity between BiVO_4_ and MIL–101–NH_2_. Due to the highly efficient light capture and sufficient electron traps induced by oxygen vacancies and the formation of a heterostructure, the improved separation and transportation rates of charge carriers are realized. In addition, the MIL–101–NH_2_/BiVO_4_ composite is favorable for Cr(VI) photocatalytic removal (91.2%). Moreover, FNBV–3 (Fe/Bi = 0.25) also exhibited an excellent reusability after five cycles.

## 1. Introduction

Heavy metal ions have increasingly attracted attention in wastewater pollutants [1]. Among the contaminants, the toxic hexavalent chromium ion is seriously threating for the ecosystem and poses a great carcinogen risk to human survival [2]. In this regard, the USEPAs guidelines considered that the concentration of Cr(VI) in drinking water exceeding 0.1 mg·L^−1^ may lead to negatives carcinogenic effects. At present, several available methods have been proposed to remedy the pollution, including catalytic reduction, chemical precipitation, membrane–based separation, and absorption [3]. Very recently, the removal of photocatalytic–based pollutants has become a feasible way to solve this problem as it is environmentally friendly and of a low cost. Since 1970, research on high–performance photocatalysts has been booming [4]. To date, countless explorations such as noble metals, semiconductor metal oxides, polymers, and inorganics have been used for the removal of light–driven pollutants [5,6,7].

BiVO_4_ has proven to be one of the classical photocatalysts with non–toxicity, a good stability, and a suitable band gap [8,9,10]. However, BiVO_4_ needs to surmount the low surface and displays a poor performance in photogenerated electron–hole pairs separation. Against that, regulating the properties of a photocatalytic system via optimizing the morphologies or structure of the photocatalyst has become a promising research topic. In a general way, the addition of surfactants, a high temperature, or a template are commonly applied for morphological modifications. The CTAB–assisted synthesis of the BiVO_4_ micro–tube by Ying et al. [11] showed a nearly five–fold enhancement compared to a micro–rod in photocatalytically decomposed MO (95%, 240 min) under a visible illumination. They reported that the hollow and tubular structures favored a large, specific surface area and the porous properties facilitated the removal activity. Similarly, Liu et al. [12] produced a sandwich–like BiVO_4_ sheet with the addition of surfactant PEG–1000 under 140 °C, which was nearly twice (86%, 150 min) as capable of photocatalytically removing MO as irregular BiVO_4_. Both of their exception activities can be attributed to the active sites enhancement, corresponding to the greater surface area [13]. There commonly exist some inevitable issues during use, such as: (1) surfactants may block the surface active sites; (2) surfactants are prone to residue; and (3) there may be surfactants with high levels of toxicity and low biodegradability. In addition, metal–organic frameworks (MOFs) possess a great potential in photocatalysis as the charming porous and semiconductor–like property [14,15]. Their organic linkers are natural for valence, and amine group introduction was contributes to facilitating the charge separation by the effect of a ligand–to–metal charge transfer (LMCT) [16]. Compared to other MOFs, the merits of water stability and facile preparation make MIL–101–NH_2_ an ideal catalytic candidate for wastewater purification. MIL–101–NH_2_ exhibited great promise in photocatalytic activity because of the Fe–O cluster which can cause direct excitation and induce an electron transfer [17]. Additionally, the large active surface for photogenerated charge carriers interact with the pollutant is also help in the promotion [18]. All these characteristics enable MIL–101–NH_2_ to be utilized in photocatalysis. Huang et al. [19] studied the preparation of a Z–Scheme V_2_O_5_/NH_2_–MIL–101 (Fe) composite, which can perform well a photocatalytic degradation toward the removal of TC (88.3%, 120 min). Immobilizing CrPd nanoparticles over MIL–101–NH_2_, Cr_0.4_Pd_0.6_/MIL–101–NH_2_ was employed in the generation of hydrogen from formic acid at 323 K (2009 mol H_2_ mol Pd^−1^h^−1^) and was much more superior than the vast majority of noble metal catalysts reported [20]. Nonetheless, MIL–101–NH_2_ is still limited in its actual application due to the expensive cost. 

Herein, by combining the advantages between BiVO_4_ and MIL–101–NH_2_, we have surprisingly obtained the flower–like MIL–101–NH_2_/BiVO_4_ composite without surfactants. The three–dimensional structure facilitates the improved absorption of visible light. Otherwise, the preparation was facile and free of surfactants. In this hybrid, the stability of the composite promoted through the new connection was constituted as –COO^−^–Bi^3+^ bond. The separation of photogenerated electron–hole pairs are fostered with the construction of the built–in electric field and it enhances the photocatalysis under a visible illumination. In this work, the characterization of MIL–101–NH_2_/BiVO_4_ was proposed and the appropriate experimental condition of the photocatalytic reduction of Cr(VI) was optimized.

## 2. Results and Discussion

### 2.1. Materials Characterization

**Scheme 1:**
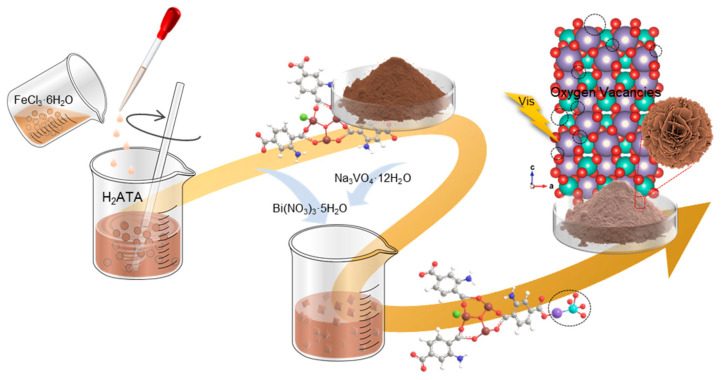
Schematic illustration of the MIL–101–NH_2_/BiVO_4_ composite’s construction.

Following the method illustrated in Scheme 1, the construction of the MIL–101–NH_2_/BiVO_4_ composite was achieved and the product is yellowish–brown in color. The pre–prepared addition of MIL–101–NH_2_ drove the self–assembly of BiVO_4_ into a flower–like structure through a facile hydrothermal method. Additionally, FNBV–3 was chosen for the materials’ characterization, outlined below, based on its impressive performance in the photocatalytic reduction of Cr(VI).

**Figure 1:**
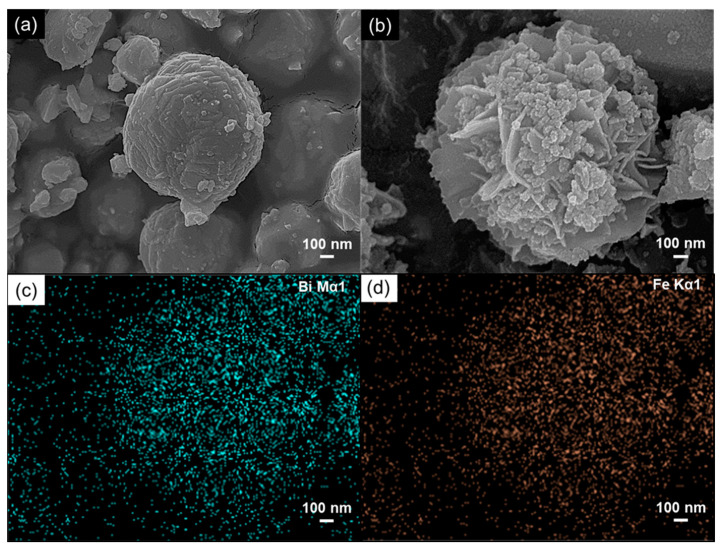
SEM images of BV (**a**) and FNBV–3 (**b**); SEM–EDS elemental mapping of FNBV–3; (**c**) Bi and (**d**) Fe.

SEM studies were utilized to lay bare the morphology and microstructure of the as–prepared samples (Figure 1). It can be clearly observed from Figure 1a that bare BiVO_4_ particles have a sphere–like structure. The pristine MIL–101–NH_2_ was well–organized octahedral in shape with a 100 nm diameter (Figure S1a). As disclosed in Figure 1b, flower–like FNBV–3 was assembled by BiVO_4_ nanosheets and embedded with MIL–101–NH_2_ intimately, which is helpful for charge carriers and a mass transfer. The structure of the flower–like composite becomes more pronounced in Figure S1b as there is less MIL–101–NH_2_ content in FNBV–1, and becomes overloaded in FNBV–7 (Figure S1c). Microcosmic appearance changes upon the complex are credited to the electrostatic attractions of the ions and hydrophobicity groups (–COOH). Predictably, Bi^3+^ ions were bonded to Fe–MOF to reinforce the interaction as well as to control the growth and nucleation rate of BiVO_4_. A similar situation was confirmed in the study from Malathi et al. [21]. In addition, the elemental mapping analysis of FNBV–3, depicted in Figure 1c,d, reveals that the elemental distribution of Bi and Fe was both unambiguously included in the position, while Bi and Fe were taken for the characteristic element of BiVO_4_ and MIL–101–NH_2_ to disclose the distribution of the two components, respectively. For a further confirmation, ICP–OES was detected to investigate the actual molar ratio of Fe/Bi in the sample. The actual value of Fe/Bi in the acquired FNBV–3 (0.23) is approximated compared to the nominal amount. As revealed in Figure S2, the real introduction of the MIL–101–NH_2_ (34.8%) and BiVO_4_ (65.2%) component in FNBV–3 was assessed by TG curve analysis. The BET specific surface area promotion is demonstrated in Figure S3 and strongly proves the existence of MIL–101–NH_2_.

**Figure 2:**
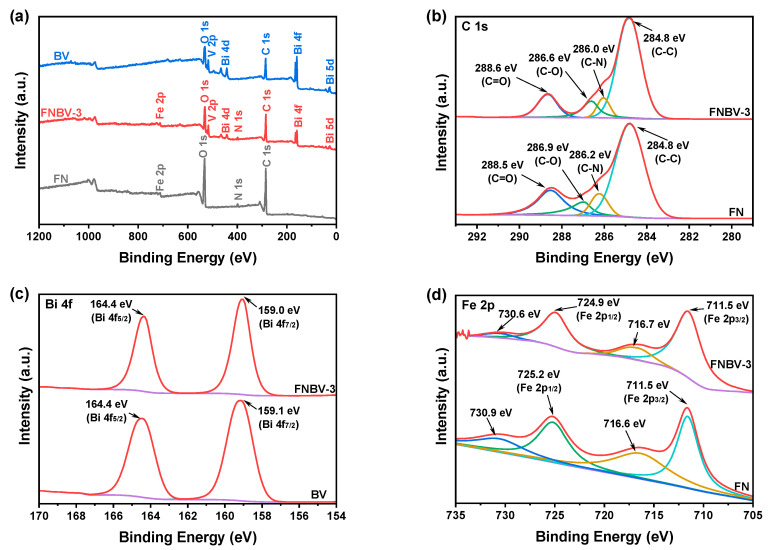
XPS spectra of BV, FN, and FNBV–3 samples: (**a**) survey scan; (**b**) C 1s; (**c**) Bi 4f; (**d**) Fe 2p spectra.

The XPS analysis of the material is summarized in Figure 2. The relevant peaks were calibrated with the C 1s signal of contaminant carbon and have been labeled on the side, respectively [22]. Figure 2a depicts the XPS typical survey spectrum investigating the existence of Bi, V, O, C, N, and Fe elements in the FNBV–3 composite. Additionally, both elements were consistent with two monomer samples. Supported by the same circumstance in the study from Sun [23], the lattice oxygen intensity in the O 1s XPS spectra decreased with the lack of the O atom and the presence of oxygen vacancies for FNBV–3, as shown Figure S4. The high–resolution orbital scan for C 1s in Figure 2b exhibited binding energies at 284.8, 286.0, 286.6, and 288.6 eV. These peaks can be associated with C–C, C–N, C–O, and C=O bonds, confirming the presence of benzoic rings and H_2_ATA linkers in FNBV–3. Contrasted with the corresponding peaks of pure FN, C=O shifted towards the positive side, which may be due to the charge density promotion over C=O after the protonation of FN [24]. As presented in Figure 2c, two typical signals for Bi 4f_7/2_ and Bi 4f_5/2_, a consensus among the previous theory, were resolved into 159.0 and 164.4 eV, which represented the characteristic of the Bi^3+^ state. The opposite shifted compared with the pristine over bismuth and C=O, suggesting there is an electrovalent bonding force between Bi^3+^ and –COO^−^ [25]. BV can serve as a charge donor, and the electrons were transferred to FN in the FNBV–3 system. Consequently, it is recommended that FN to BV pose an efficient charge migration for the formation of a heterojunction between the two components. Similar circumstances have arisen in Zhao’s paper [26]. In addition, 711.5 and 724.9 eV in the Fe 2p spectrum can be ascribed to Fe 2p_3/2_ and Fe 2p_1/2_, with satellite peaks being located at 716.7 and 730.6 eV (Figure 2d). These peaks can manifest the presence of iron (III) oxide in FNBV–3 [16]. Consequently, the outcome of XPS provides adequate support for the constitution of the MIL–101–NH_2_/BiVO_4_ compound via the hydrothermal method. 

**Figure 3:**
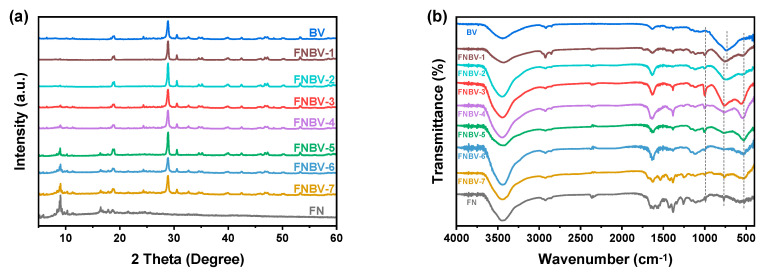
(**a**) XRD patterns of FN, BV, FNBV–n (*n =* 1–7) samples; (**b**) FT–IR spectra of FN, BV, FNBV–n (*n =* 1–7).

XRD patterns (Figure 3a) were determined to ascertain the formation and crystallographic structure of the MIL–101–NH_2_/BiVO_4_ composite. The diffraction peaks of FN were consistent with the reference, evidencing the successful synthesis of MIL–101–NH_2_ [27]. Confirming the effective preparation of pure BiVO_4_, BV displaying narrow line width peaks can be indexed to the high crystallinity monoclinic BiVO_4_ (JCDPDS Card No. 83–1699) [22,28]. Moreover, the detection of any peaks can be ascribable to other phases or impurities. For FNBV–n, the characteristic signal coexistence strongly inherits with those of FN and BV compounding when *n* = 3–7. The low content and high distribution of FN may lead to FNBV–1 and FNBV–2 being undetectable. Accordingly, the successful complexation of FN and BV was thus suggested, and oxygen vacancies show a negligible influence on the hybrids of MIL–101–NH_2_ and BiVO_4_.

To determine the constitution of the functional group, FT–IR patterns were conducted in Figure 3b. Typically, the stretching and bending vibration of the O–H bond at around 3430 cm^−1^ and 1630 cm^−1^ in all the spectra is assigned to the presence of absorbed H_2_O molecules. For FN, the characteristic absorption of 528 cm^−1^ is associated with Fe–O. Furthermore, peaks at 1257 cm^−1^ and 2900 cm^−1^ are attributed to C–N and –NH_2_ in the linker of the framework [29]. The absorption of 744cm^−1^ took place in the BV spectrum in respect of the V–O stretching vibration peak and confirmed the observation of monoclinic scheelite BiVO_4_, which was synthesized from XRD [30]. Seen from the FNBV–n spectra, the presence of MIL–101–NH_2_ and BiVO_4_ was distinctly proved. Thereby, the introduction of oxygen vacancies cannot destroy their chemical structure’s stability. The slight red shift of the V–O bond in FNBV–n accounts for a length increase and the Raman spectra were utilized to prove it [31,32]. As shown in Figure S5, the peak position of υ_as_(V–O) shifted towards the lower wavelength region with MIL–101–NH_2_. This further supports that the V–O bond length of BiVO_4_ increased corresponding to the empirical relation below [33]:

υ = 21349·e^−1.9176·R^ (1)

where υ is the location of the stretching mode and R is related to the length of the bond.

### 2.2. Photocatalytic Behaviours on Cr(VI) Reduction

**Figure 4:**
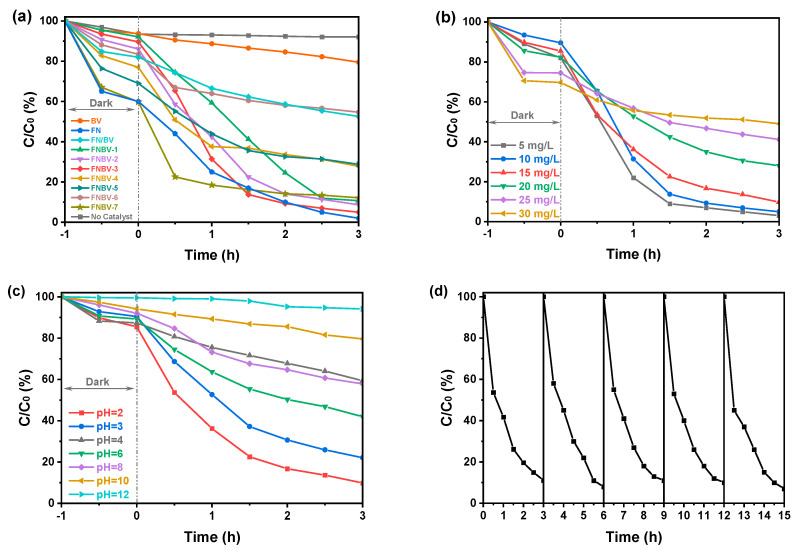
(**a**) Photocatalytic reduction of 10 mg·L^−1^ Cr(VI) solution via the preparation; effect of Cr(VI) concentration (**b**) and pH value (**c**) on the reduction over FNBV–3; (**d**) recycling test for photocatalytic reduction of 15 mg·L^−1^ Cr(VI) solution. Reaction conditions: 1g·L^−1^, λ > 420 nm, room temperature.

The Cr(VI) PCR over different materials was performed on the K_2_Cr_2_O_7_ solution. Before it was exposed to an illumination, a dark adsorption in 1 h was set for the adsorption and desorption equilibrium achievement. The blank examination was operating as well, assuring that the spontaneously Cr(VI) depoisonous under irradiation without catalysts, and a direct reaction with Cr(VI) in the absence of light as well. Figure 4a shows the removal efficiency of several products in Cr(VI)–containing solution (10 mg·L^−1^). It is clear that the elimination capacity was increased after BV coupled with FN and that the enhancement of the photocatalytic activity was consistent with DRS. Such an enhancement may be related to the factual accessible active site. For FNBV–n (*n =* 1–3) could be bound up with the high separation of photogenerated carriers while overloading may block the transfer [34]. Meanwhile, the promotion can also be related to oxygen vacancies. It has shown a capability in trapping electrons and the obstacle effect for a photogenerated recombination. The adsorption performance of FNBV–7 was similar to FN. This phenomenon may be explained by the excessive coverage of FN, served as a kind of aggregation and hindering the irradiation’s penetration and absorption [35]. Among catalysts, FNBV–3 demonstrated an outstanding PCR efficiency (95.0%) compared to BV (20.5%) and the FN/BV physical mixture (FN: 28.1 mg, BV: 13.9 mg; 47.5%). Hence, the potent heterostructure between FN and BV was fabricated successfully; that is, it was valid in virtue of the promotion in the PCR. Meanwhile, oxygen vacancies which are capable of capturing light also contributed. At the same time, Cr(VI) with tap water was carried out under the uniform experimental conditions to ensure the result of the coexisting ions (95.5%). It is also noted that the superior adsorption of FN contributed to the similar removal capacity with FNBV–3. However, the expensive cost and the worse photoreaction rate were the key to explain its limitation, nonetheless. Additionally, the reaction kinetic study was acquainted by the pseudo–first–order (PFO) (Equation (2), Figure S6a,b) and pseudo–second–order (PSO) (Equation (3), Figure S6c,d), respectively [36,37]. In agreement with previous reports, the correlation kinetic curves fitted well with the Langmuir–Hinshelwood (L–H) first–order model [38] as a coefficient (R_1_^2^ for PFO and R_2_^2^ for PSO), which alluded to the fitness of the models with the relative data obtained. The highest value of κ_1_ was obtained from FNBV–3, which was about 20–fold compared to that of BV. Moreover, the synergistic factor *sκ* (Equation (4)) for FNBV–3 is applied for quantitatively appraised the synergistic effect [36]. The value of 2.6 revealed an interaction enhancement between the two components for the photocatalytic activities. Above, FNBV–3 was consequently chosen for the following investigation.

−ln(C/C_0_) = *κ*_1_t (2)

1/C = *κ*_2_t + 1/C_0_ (3)

*Sκ* = *κ*/(x·*κ_BV_* + y·*κ*_FN_) (4)

where C_0_ (mg·L^−1^) is the initial concentration of the Cr(VI) solution and C (mg·L^−1^) is that at time t; *κ*_1_ (h^−1^) is the rate of reduction given as the slope in PFO while PSO is the rate constant for *κ*_2_ (L·h·mg^−1^); and *κ*, *κ*_BV_, and *κ*_FN_ are the fitted reaction rate constants of FNBV–3, BV, and FN, respectively.

The effect of the initial concentration study was unveiled in Figure 4b. Evidently, the total reduction efficiency was gradually inclined to deteriorate as the concentration was increasing, which may be influenced by system with the lack of photo–induced active substances. In addition, the surface of the photocatalyst accumulated by the saturation of Cr(VI) can also be detrimental to the performance. Given that FNBV–3 exhibited a similarity in terms of the removal result in 10 and 15 mg/L (95.0%, 91.2%), the latter allowed for obtaining the optimal one in the present study after measuring the removal capacity (9.5 and 13.7 g·mg^−1^). 

It is noteworthy to mention that the reaction system’s pH value was considered to be a crucial parameter in the reduction of Cr(VI) [39]. Figure 4c and Figure S8 disclosed the photocatalytic reduction of Cr(VI) with different pH value gradients. The photoreduction property declined as the pH value increased, which may be because of the existing form of Cr(VI) and the surface potential of the catalyst. The zeta potential was tested and is plotted in Figure S9. The surface of FNBV–3 was covered and positively charged above 3.1 as the zero–point charge (pH_ZPC_) pointed to ca. 3.1. This can explain why FNBV–3 performed best when adopted as pH = 2 with the electrostatic attraction effect between FNBV–3 and Cr_2_O_7_^2−^ or HCrO_4_^−^ (the dominating species of Cr(VI) in an acidic aqueous dispersion) [40]. The abundant H^+^ surroundings are a great help in the detoxification of Cr(VI) as well. The photocatalytic activity of FNBV–3 under the optimal condition was compared to other BiVO_4_–based catalysts reported with a different morphology in previous literatures, as tabulated in Table S1. 

**Figure 5:**
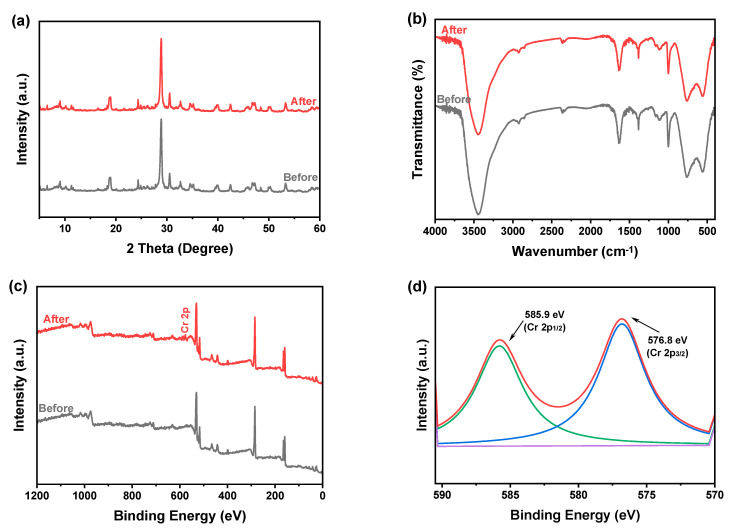
XRD (**a**) and FT–IR (**b**) spectra of fresh and used FNBV–3; XPS spectra of the used FNBV–3 samples; (**c**) survey scan; (**d**) Cr 2p spectra.

According to Figure S10, the removal capability of Cr(VI) without visible light was attribute to the adsorption of Cr(VI) over FNBV–3. Furthermore, a subsequent reusability assessment played a vital role in the industrial application estimate. As illustrated in Figure 4d, the recovered can retain a superior reduction quality after five cycles. For the used FNBV–3, the XRD (Figure 5a) and FTIR (Figure 5b) results argued that there is no contravention with the new one and they provide evidence of the composition being self–steady. XPS analysis (Figure 5c,d) was applied to monitor the valence state of the Cr element on the surface of the sample used. There are two discernible peaks supporting the existence of Cr(III) which appeared in the high–resolution spectrum of Cr 2p [41]. It can be deduced that the poisonous Cr(VI) can convert to harmless Cr(III) with the help of visible light and FNBV–3. The ICP detection confirmed that the lixiviating of Fe^3+^ in the reaction solution was negligible. Such can be presumed as the as–prepared sample was a perfect match with the recyclability and stability in real photocatalysis. Thus, MIL–101–NH_2_/BiVO_4_ composite can serve as an appropriate photocatalyst to solve the pollution of heavy ions Cr(VI) in water.

**Figure 6:**
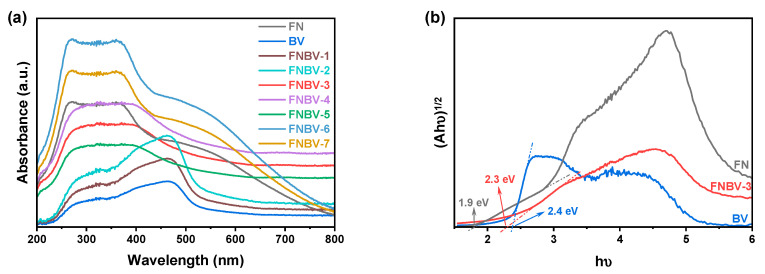
UV–vis diffuse reflectance spectra of all samples (**a**) with (Ahυ)^1/2^ vs. hυ curves (**b**).

UV–vis DRS was measured to access the optical absorption quality and band gap energy (E_g_, eV) of the samples. As demonstrated in Figure 6a, the absorption threshold was red shifted to the visible region (about 700 nm) in BiVO_4_–based hybrids, interpreting the substantially boosted photocatalytic reduction [42]. The Tauc plot method is defined in Equation (5) and is calculated for the E_g_ evaluation in Figure 6b [43].

(ɑhυ)^1/n^ = A·(hυ − E_g_) (5)

where ɑ and hυ are the absorption coefficient and the photon energy, respectively. The value of n was linked to the characteristic direct or indirect transition in the semiconductor. A represents a constant.

The values of E_g_ for FN, BV, and FNBV–3 were estimated to be 1.9, 2.4, and 2.3 eV. Collectively, FNBV–3 was accompanying by a narrow band gap as well as a sensitivity to visible light. To some extent, the addition of FN brings an intimate heterointerface to BV, which endows a great facilitator for competence in the spatial separation of photogenerated electron–hole pairs [44].

### 2.3. Mechanism on the Photocatalytic Efficiency Promotion

On the basis of previous research papers, PCR was processed with the contribution of multitudinous reactive substances. To identify the major active specie responsible for the Cr(VI) reduction over FNBV–3, control experiments with a few scavengers are executed in Figure S11 [45]. Among the radical quenching research, AgNO_3_ (0.2 mmol·L^−1^), Ethylenediaminetetraacetic acid disodium (EDTA–2Na, 0.2 mmol·L^−1^), p–benzoquinone (BQ, 0.2 mmol·L^−1^), and tertiary butanol (TBA, 0.2 mmol·L^−1^) were selected to consume the electronics (e^−^), holes (h^+^), superoxide anion radicals (·O_2_^−^), and hydroxyl radicals (·OH), respectively. This weakened the removal rate with the trapping of AgNO_3_, deducing that e^−^ led a predomination in the photocatalytic process, while the capture of O_2_^−^ or ·OH could also contribute to the drop. The influence caused by the TBA should be ascribed to the equilibrium transmitting to Cr(VI) [46,47]. On the contrary, the removal promotion with the addition of EDTA–2Na may correlated to the enhanced separation of the photogenerated pairs and the increase in the e^−^ [48]. 

**Figure 7:**
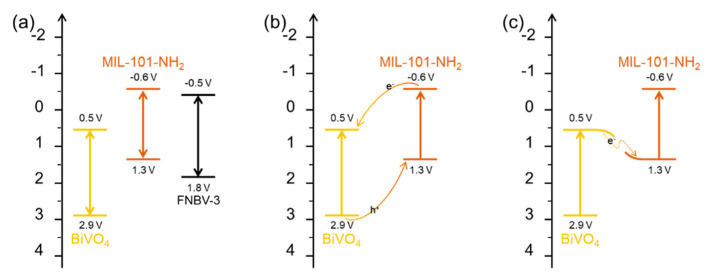
(**a**) Band structures of MIL–101–NH_2_/BiVO_4_; schematic diagram of (**b**) traditional and (**c**) Z–scheme heterojunction pathway for carrier separation.

In light of the estimated band structure, a conceivable mechanism for the improvement of the photoreduction manifestation in FNBV–3 is suggest in Figure 7 tentatively [49]. As is well known, the composite proposed a type–II staggered band alignment, which is in a condition that the heterojunction interface between MIL–101–NH_2_ and BiVO_4_ was followed by the conventional separation of photogenerated electron–hole pairs. The O_2_^−^ and ·OH could not form, which conflicted with the quenching test, as the band was located at the position which was either more positive or negative than E(O_2_/O_2_^−^) and E(H_2_O/·OH) [46,50,51]. Therefore, carrier transfers do obey with the Z–scheme heterojunction model. The aggregate was photogenerated at the CB of MIL–101–NH_2_ and VB of BiVO_4_, so that FNBV–3 could generate O_2_^−^ and ·OH under irradiation.

**Scheme 2:**
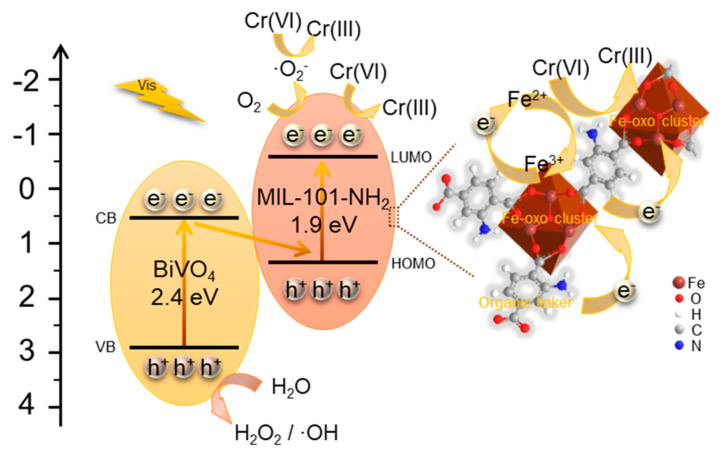
Proposed mechanism for removal of Cr(VI) over MIL–101–NH_2_/BiVO_4_ composite.

From the analysis above, in this paper we raised a plausible mechanism for the removal of Cr(VI). Before irradiation, the MIL–101–NH_2_/BiVO_4_ composite facilitated in the adsorption by a large specific surface area and the charge effect on the surface. Then, the involved photolysis after being exposed to visible light is documented in Equations (6)–(13). Based upon the aforementioned results, the direct Z–scheme heterojunction was confirmed and illustrated in Scheme 2, which was consistent with the previous correlated literature [52].

(6) $$\text{FNBV–3}\overset{h\upsilon }{\to }h^{+}+e^{-}$$

O_2_ + e^−^ → ·O_2_^−^ (7)

(8) $${\mathrm{Cr}}_{2}{O_{7}}^{2-}+\cdot O_{2^{-}}\overset{h\upsilon }{\to }2{\mathrm{Cr}}^{3+}+O_{2}$$

Cr_2_O_7_^2−^ + 14H^+^ + 6e^−^ → 2Cr^3+^ + 7H_2_O (9)

2H_2_O + 2h^+^ → H_2_O_2_ + H^+^ (10)

Cr_2_O_7_^2−^ + 3H_2_O_2_ + 8H^+^ → 2Cr^3+^ + 7H_2_O + 3O_2_ (11)

Fe^3+^ + e^−^_(CB)_ → Fe^2+^ (12)

Fe^2+^ + Cr_2_O_7_^2−^ → Fe^3+^ + Cr^3+^ (13)

## 3. Experimental

### 3.1. Material

The chemical reagents used were all commercially available without further purification and the corresponding specific information is outlined in the Supplementary Materials.

### 3.2. Synthesis of MIL–101–NH_2_/BiVO_4_ Composite

The MIL–101–NH_2_ powder was prepared via a modified facile solvothermal method, as has been previously documented in the literature, and was denoted as FN [17,53]. The MIL–101–NH_2_/BiVO_4_ composites with varying molar ratios were synthesized through a hydrothermal method and the preparation process was illustrated in Scheme 1. At first, 0.3 mmol of Bi(NO_3_)_3_·5H_2_O and 0.02 mmol of FN were ultrasonically dispersed into 40 mL of DI. Subsequently, 0.3 mmol of Na_3_VO_4_·12H_2_O was added slowly stirred for 30 min. The above mixture was kept under 373 K for 24 h in a 100 mL Teflon–lined autoclave. After centrifugation and washing, the powder was collected. The obtained powder was placed in a vacuum oven and kept dry at 333K overnight. The final sample was nominated as FNBV–3. Likewise, FNBV–n (*n* = 1–7) composites were created with a different molar ratio of Fe to Bi (0.05, 0.10, 0.25, 0.50, 1.00, 2.00, 4.00). For comparison, the pristine BiVO_4_ was yielded under the same manner as outlined above, with the absence of MIL–101–NH_2_, and was referred to as BV.

### 3.3. Characterization

Scanning electron microscope (SEM) images (JSM–7600F) were used to obtain the morphology and structure of the product. Meanwhile, the energy–dispersive X–ray spectroscopy (EDS) of the composite was recorded on INCA X–Act, Oxford Instruments. The gas adsorption isotherm and specific surface area were deduced on the basis of Brunauer Emmett Teller (BET) methods (BSD–PM2). Moreover, XRD patterns were conducted by an Ultima IV equipped with Cu Kα radiation, and the crystal data and phase structure were analyzed. The chemical states and the composition of the elements on the surface can be detected through X–ray photoelectron spectroscopy (XPS) (AXIS UltraDLD). VERTEX 80v, referring to a KBr disk, was employed to document the Fourier transform infrared spectroscopy (FT–IR). The UV–vis absorption spectra were acquired on a UV–2802 spectrometer. The UV–vis diffuse reflection spectra (UV–vis DRS) of the specimens were acquired by using UV–2600 with BaSO_4_ performed as has been documented.

### 3.4. Photocatalytic Reduction of Cr(VI)

The photocatalytic reduction (PCR) activity on Cr(VI) under visible light was systematically investigated at room temperature. The catalytic reaction was performed through a Xe lamp equipped with a 420 nm cut–off filter (CEL–HXF300, 300 W light intensity: 100 mW·cm^−2^). Before illumination, the as–prepared product was added into Cr(VI) solution (1 g·L^−1^, 15 mg/L) and was stirred in the dark for 1 h until it reached an adsorption–desorption equilibrium. During the process, the reaction samples (each in 3 mL) were filtered (0.22 μm syringe filter) at each interval of time and the filtrate concentrations were monitored by the purple complex absorbance at 540 nm, according to the 1,5–diphenylcarbazide colorimetric (DPC) method [17]. Data were averaged for each sample after triple–testing to determine the result. In addition, the same reduction experiment was carried out five times with the used material being gathered to ensure the stability of the as–prepared composite, as well as a dark adsorption in a 12 h study. The PCR efficiency of Cr(VI) was evaluated based on η = (C_0_ − C_t_)/C_0_ × 100%, where η refers to the removal ability and C_0_ and C_t_ represents the solution concentration before and after the experiment. 

A necessity is obtaining the band structure of the semiconductor to fully investigate the separation behavior of the heterostructures. The potential of the value band (VB) and conduction band (CB) component were estimated by the empirical formulas [22,54]:

E_VB_ = X + 0.5E_g_ + E^e^ (14)

X = [x(A)^a^ x(B)^b^ x(C)^c^ x(D)^d^]^1/(a + b + c + d)^ (15)

E_CB_ = E_VB_ − E_g_ (16)

where E_VB_ and E_CB_ correspond to the VB and CB edge position, respectively; X is the electronegativity of the semiconductor; E_g_ relates to the band–gap energy; and E^e^ represents the energy of free electrons vs. hydrogen (4.5 eV). In the report, the above equations (Equations (14)–(16)) were the value of VB and CB from BiVO_4_. Based on the band gap of MIL–101–NH_2_ and BiVO_4_, the mechanism which refers to the MIL–101–NH_2_/BiVO_4_ composite efficiently and photocatalytically reducing Cr(VI) is exhibited in Scheme 2.

## 4. Conclusions

In summary, flower–like MIL–101–NH_2_/BiVO_4_ composites with varying molar ratios of Fe/Bi were successfully prepared and characterized, with the existence of oxygen vacancies and the newly formed interaction between –COO^−^ in MIL–101–NH_2_ and Bi^3+^ in BiVO_4_. The as–synthesized materials were evaluated by the reduction of the hexavalent chromium under a visible–light illumination. Fe/Bi = 0.25 (FNBV–3) was selected for the optical molar ratio according to its photocatalytic performance (15 mg/L, pH = 2, 91.2%, 3 h). It could be reasonable to infer that the modification of BiVO_4_ with MIL–101–NH_2_ enhanced the light–harvesting ability and accelerated the migration of the photogenerated carrier, contributing to the prominent photocatalytic activity. Moreover, the material also bared an outstanding stability and recyclability. This study announced the feasible utilization in detoxifying Cr(VI) under the co–effect of a light irradiation and MIL–101–NH_2_/BiVO_4_ composite. With this work, we hope to share some insight and reflect on the recent photocatalytic practices.

## Data Availability

The data will be available upon reasonable request.

## Supplementary Materials

The following supporting information can be downloaded at: https://www.mdpi.com/article/10.3390/molecules28031218/s1, Figure S1: SEM image of (a) FN; (b) FNBV–1 and (c) FNBV–7; Figure S2: TG curves of BV, FN and FNBV–3; Figure S3: N_2_ adsorption–desorption isotherms of FN, BV and FNBV–3; Figure S4: O 1sXPS spectra of BV and FNBV–3 samples; Figure S5: Raman spectra of FN, BV and FNBV–3; Figure S6: The corresponding (a,b) pseudo–first–order and (c,d) pseudo–second–order reaction kinetic linear simulation curves; Figure S7: The corresponding first–order Langmuir–Hinshelwood model of reaction kinetic study; Figure S8: The corresponding first–order Langmuir–Hinshelwood model of reaction kinetic study; Figure S9: Zeta Potential measurement of BV and FNBV–3; Figure S10: The removal performance of FNBV–3 in dark; Figure S11: Influence of scavengers in the performance of FNBV–3 composite; Table S1: Comparison of various photocatalysts efficiency in pollutants removal.
