# Supplementary material for: BiVO4–Deposited MIL–101–NH2 for Efficient Photocatalytic Elimination of Cr(VI)
Source: Molecules. 2023 Jan 26;28(3):1218. doi: 10.3390/molecules28031218 (PMC9921149; doi:10.3390/molecules28031218)
Supplement: Supplementary file 1 [file molecules-28-01218-s001.zip › molecules-2151094-supplementary.pdf]

**Supplementary information**

# $BiVO_4$–deposited MIL–101–$NH_2$ for efficient photocatalytic elimination of Cr(VI)

*Huiwen Sun* [a], *Qihang Dai* [a], *Ju Liu* [a], *Tiantian Zhou* [a], *Muhua Chen* [a], *Zhengchun Cai* [a], *Xinbao Zhu* [a], *Bo Fu* [a,] *

[a] Jiangsu Co–Innovation Center of Efficient Processing and Utilization of Forest Resources, Jiangsu provincial key lab for the chemistry and utilization of agro–forest biomass, College of Chemical Engineering, Nanjing Forestry University, Nanjing 210037, China.

*Material*

Bismuth nitrate pentahydrate ($Bi(NO_3)_3 \cdot 5H_2O$, 99%), Ammonium Metavanadate ($NH_4VO_3$, 99%), Sodium Orthovanadate Dodecahydrate ($Na_3VO_4 \cdot 12H_2O$, 99%), Nitric acid ($HNO_3$, 65-68%, Sinopharm Chemical Reagent Co., Ltd.), 2-Aminoterephthalic acid ($H_2ATA$, 98%), Titanium isopropoxide (TTIP, 95%), Ferric Chloride Hexahydrate ($FeCl_3 \cdot 6H_2O$, ≥99%, Sinopharm Chemical Reagent Co., Ltd.), Methanol (MeOH, ≥99.8%), and N,N–dimethylformamide (DMF, ≥99.8%) were supplied by Shanghai Macklin Biochemical Co., Ltd. and utilized as received without further treatment.

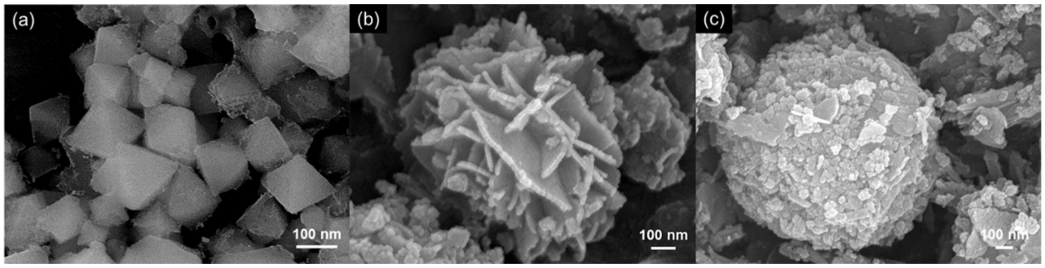


**Figure S1.** SEM image of (a)FN; (b)FNBV–1 and (c)FNBV–7.

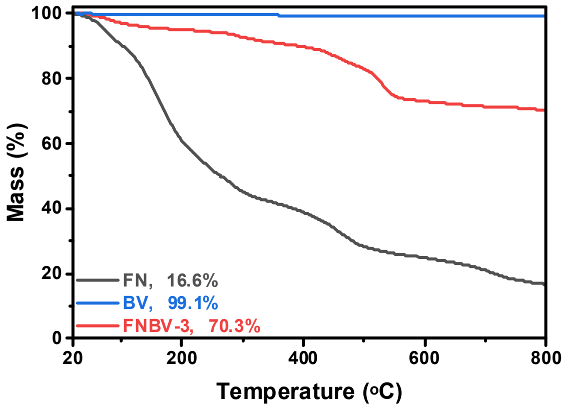


**Figure S2.** TG curves of BV, FN and FNBV–3.

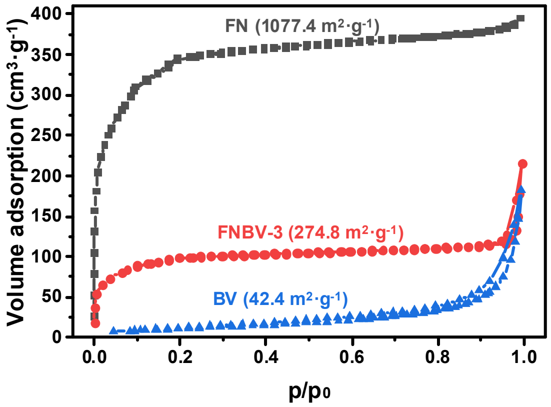


**Figure S3.** $N_2$ adsorption-desorption isotherms of FN, BV and FNBV–3.

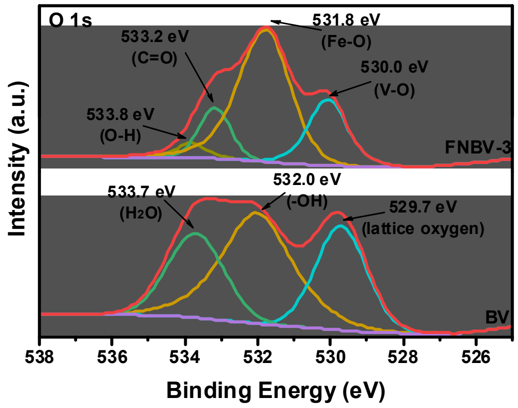


**Figure S4.** O 1sXPS spectra of BV and FNBV–3 samples.

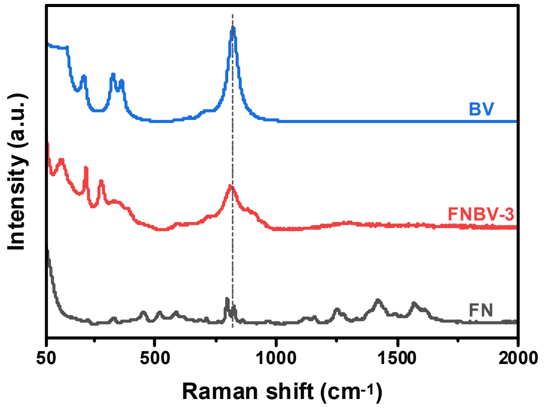


**Figure S5.** Raman spectra of FN, BV and FNBV–3.

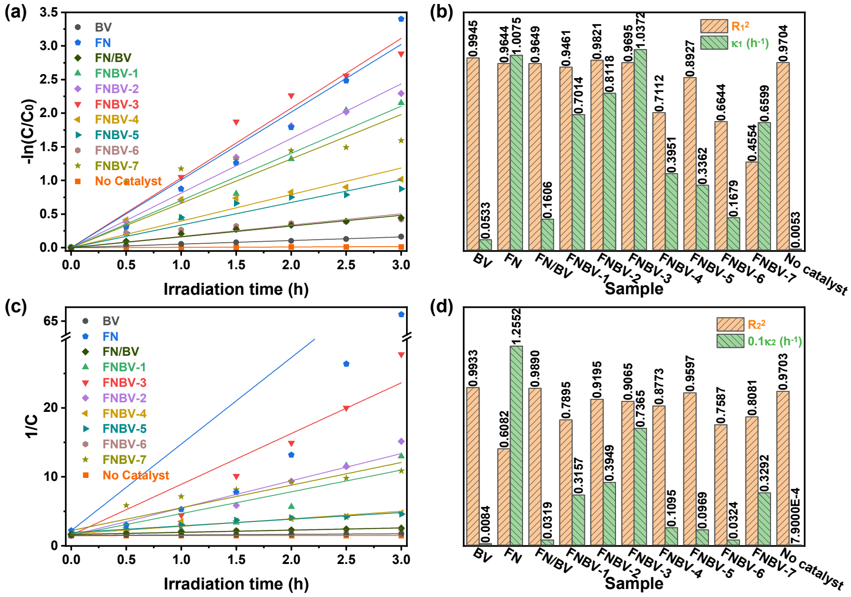


**Figure S6.** The corresponding (a, b) pseudo–first–order and (c, d) pseudo–second–order reaction kinetic linear simulation curves.

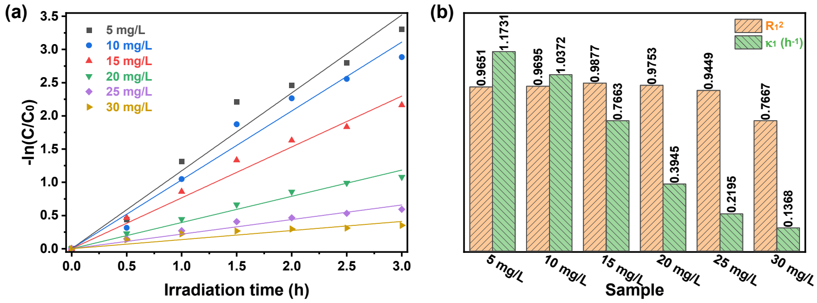


**Figure S7.** The corresponding first–order Langmuir–Hinshelwood model of reaction kinetic study.

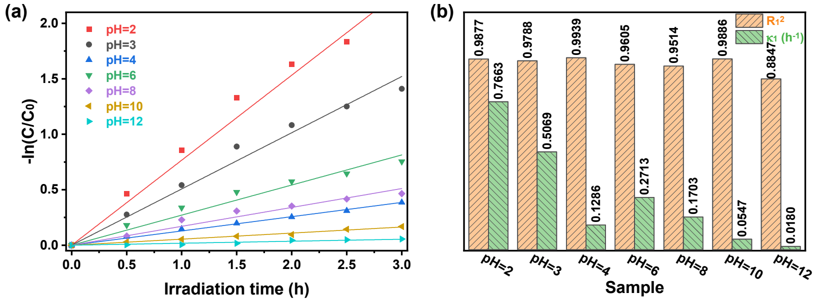


**Figure S8.** The corresponding first–order Langmuir-Hinshelwood model of reaction kinetic study.

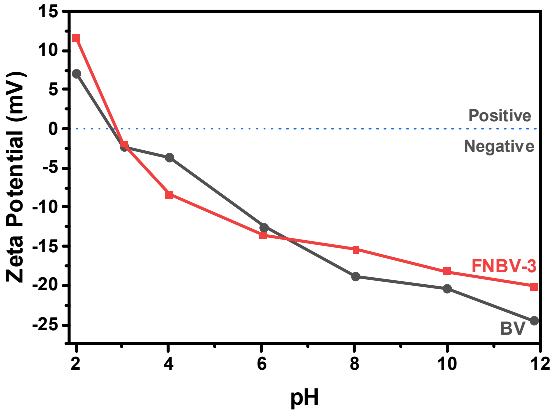


**Figure S9.** Zeta Potential measurement of BV and FNBV–3.

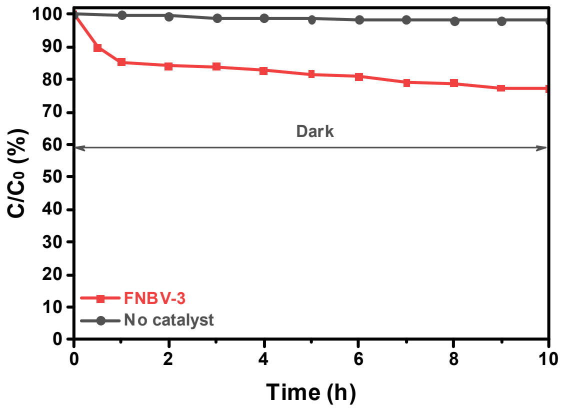


**Figure S10.** The removal performance of FNBV–3 in dark.

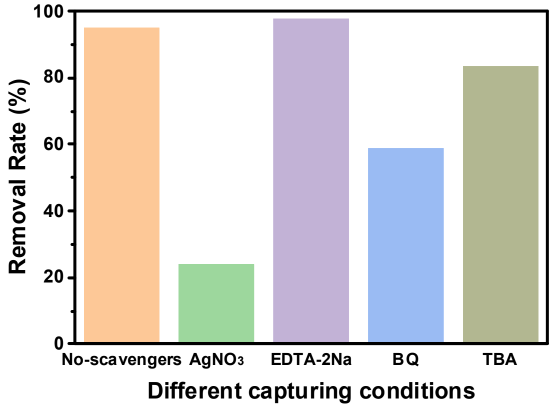


**Figure S11.** Influence of scavengers in the performance of FNBV–3 composite.

**Table S1.** Comparison of various photocatalysts efficiency in pollutants removal.

| Catalysts | Morphology | Removal efficiency (%) | Catalytic conditions | Reference |
|---|---|---|---|---|
| MIL–-101–$NH_2/BiVO_4$ | Flower-like | 91.2 | 180 min, 1 g·$L^{-1}$ 15 mg·$L^{-1}$ Cr(VI) | This work |
| $BiVO_4$–$Fe_3O_4$ | Irregular | 87.1 | 120 min, 5 g·$L^{-1}$ 5 mM/L CA 5 mg·$L^{-1}$ Cr(VI) | [1] |
| $Fe_3O_4/BiVO_4/CuS$ | Irregular | 78.8 | 90 min, 1 g·$L^{-1}$ 10 mg·$L^{-1}$ Cr(VI) | [2] |
| $BiVO_4@MoS_2$ | Core-shell structure | 76.5 | 90 min, 0.4 g·$L^{-1}$ 15 mg·$L^{-1}$ Cr(VI) | [3] |
| Ce MOF/mc $BiVO_4$ | Irregular | 74.7 | 180 min, 0.1 g·$L^{-1}$ 10 mg·$L^{-1}$ MO | [4] |
| Er–-$BiVO_4$ | Near-nuts | 84 | 180 min, 0.5 g·$L^{-1}$ 10 mg·$L^{-1}$ MO | [5] |
| $Cu_2O/BiVO_4$ | Plate-like | 73 | 150 min, 0.5 g·$L^{-1}$ $2\times10^{-5}$ mol·$L^{-1}$ MO | [6] |
| $AgVO_4/BiVO_4$ | Flower-like | 74.9 | 150 min, 0.4 g·$L^{-1}$ 15 mg·$L^{-1}$ Cr(VI) | [7] |
| Ag/AgBr/$BiVO_4$ | Irregular | 91.7 | 60 min, 1 mM EDTA 10 mg·$L^{-1}$ Cr(VI) | [8] |

## Reference


1. Ke, T.; Guo, H.; Zhang, Y.; Liu, Y. Photoreduction of Cr(VI) in water using $BiVO_4$-$Fe_3O_4$ nano-photocatalyst under visible light irradiation. *Environ. Sci. Pollut. Res.* **2017**, *24*, 28239-28247, doi:https://doi.org/10.1007/s11356-017-0255-0.
2. Xu, G.; Du, M.; Zhang, J.; Li, T.; Guan, Y.; Guo, C. Facile fabrication of magnetically recyclable $Fe_3O_4$/$BiVO_4$/CuS heterojunction photocatalyst for boosting simultaneous Cr(VI) reduction and methylene blue degradation under visible light. *J. Alloys Compd.* **2022**, *895*, 162631, doi:https://doi.org/10.1016/j.jallcom.2021.162631.
3. Zhao, W.; Liu, Y.; Wei, Z.; Yang, S.; He, H.; Sun, C. Fabrication of a novel p–n heterojunction photocatalyst n-$BiVO_4$@p-$MoS_2$ with core–shell structure and its excellent visible-light photocatalytic reduction and oxidation activities. *Appl. Catal. B* **2016**, *185*, 242-252, doi:https://doi.org/10.1016/j.apcatb.2015.12.023.
4. Kuila, A.; Saravanan, P.; Routu, S.; Gopinath, P.; Jang, M.; Wang, C. Improved charge carrier dynamics through a type II staggered Ce MOF/mc $BiVO_4$ n-n heterojunction for enhanced visible light utilisation. *Appl. Surf. Sci.* **2021**, *553*, 149556, doi:https://doi.org/10.1016/j.apsusc.2021.149556.
5. Moscow, S.; Kavinkumar, V.; Sriramkumar, M.; Jothivenkatachalam, K.; Saravanan, P.; Rajamohan, N.; Vasseghian, Y.; Rajasimman, M. Impact of Erbium (Er) and Yttrium (Y) doping on $BiVO_4$ crystal structure towards the enhancement of photoelectrochemical water splitting and photocatalytic performance.

*Chemosphere* **2022**, *299*, 134343, doi:https://doi.org/10.1016/j.chemosphere.2022.134343.

6. Yuan, Q.; Chen, L.; Xiong, M.; He, J.; Luo, S.L.; Au, C.T.; Yin, S.F. $Cu_2O/BiVO_4$ heterostructures: synthesis and application in simultaneous photocatalytic oxidation of organic dyes and reduction of Cr(VI) under visible light. *Chem. Eng. J.* **2014**, *255*, 394-402, doi:https://doi.org/10.1016/j.cej.2014.06.031.
7. Zhao, W.; Feng, Y.; Huang, H.; Zhou, P.; Li, J.; Zhang, L.; Dai, B.; Xu, J.; Zhu, F.; Sheng, N.; et al. A novel Z-scheme $Ag_3VO_4/BiVO_4$ heterojunction photocatalyst: Study on the excellent photocatalytic performance and photocatalytic mechanism. *Appl. Catal. B* **2019**, *245*, 448-458, doi:https://doi.org/10.1016/j.apcatb.2019.01.001.
8. Chen, F.; Yang, Q.; Wang, Y.; Yao, F.; Ma, Y.; Huang, X.; Li, X.; Wang, D.; Zeng, G.; Yu, H. Efficient construction of bismuth vanadate-based Z-scheme photocatalyst for simultaneous Cr(VI) reduction and ciprofloxacin oxidation under visible light: Kinetics, degradation pathways and mechanism. *Chem. Eng. J.* **2018**, *348*, 157-170, doi:https://doi.org/10.1016/j.cej.2018.04.170.
